# Snow mold of winter cereals: a complex disease and a challenge for resistance breeding

**DOI:** 10.1007/s00122-020-03725-7

**Published:** 2020-11-22

**Authors:** Mira L. Ponomareva, Vladimir Yu. Gorshkov, Sergey N. Ponomarev, Viktor Korzun, Thomas Miedaner

**Affiliations:** 1 Laboratory of Plant Infectious Diseases, FRC Kazan Scientific Center of RAS, Ul. Lobachevskogo 2/31, Kazan, 420111 Tatarstan Russian Federation; 2 KWS SAAT SE & Co. KGaA, Grimsehlstr. 31, 37555 Einbeck, Germany; 3 State Plant Breeding Institute, University of Hohenheim, Fruwirthstr. 21, 70599 Stuttgart, Germany

## Abstract

**Key message:**

Snow mold resistance is a complex quantitative trait highly affected by environmental conditions during winter that must be addressed by resistance breeding.

**Abstract:**

Snow mold resistance in winter cereals is an important trait for many countries in the Northern Hemisphere. The disease is caused by at least four complexes of soilborne fungi and oomycetes of which *Microdochium nivale* and *M. majus* are among the most common pathogens. They have a broad host range covering all winter and spring cereals and can basically affect all plant growth stages and organs. Their attack leads to a low germination rate, and/or pre- and post-emergence death of seedlings after winter and, depending on largely unknown environmental conditions, also to foot rot, leaf blight, and head blight. Resistance in winter wheat and triticale is governed by a multitude of quantitative trait loci (QTL) with mainly additive effects highly affected by genotype × environment interaction. Snow mold resistance interacts with winter hardiness in a complex way leading to a co-localization of resistance QTLs with QTLs/genes for freezing tolerance. In practical breeding, a multistep procedure is necessary with (1) freezing tolerance tests, (2) climate chamber tests for snow mold resistance, and (3) field tests in locations with and without regularly occurring snow cover. In the future, resistance sources should be genetically characterized also in rye by QTL mapping or genome-wide association studies. The development of genomic selection procedures should be prioritized in breeding research.

## Introduction

Snow mold is a disease complex caused by several phytopathogenic fungi that affect agricultural, ornamental, and indigenous plants under snow cover at or shortly below the freezing point (Bruehl 1982). Typically, snow mold is mainly reported from the Northern Hemisphere. There are four types of snow mold diseases, all caused by soilborne fungi or oomycetes: pink snow mold (*Microdochium nivale, M. majus*), gray or speckled snow mold (*Typhula idahoensis, T. ishikariensis, T. incarnata*), snow scald (*Myriosclerotinia borealis*), and snow rot (*Pythium iwayami* and *P. okanoganense*) (Murray et al. 1999). *Microdochium nivale* (Fr.) Samuels & Hallet and *M. majus* (Wollenw.) Glynn & S.G. Edwards are the most common reasons for biotic winter crop damage (Nakajima and Abe 1996; Tronsmo et al. 2001).

The quantitative and qualitative composition of the snow mold-causing community greatly varies depending on the crop, region, and ecological conditions (Chang et al. 2006). In Russia, an analysis of 46 strains from five regions revealed that 59% of the strains were *M. nivale* and 28.3% were *M. majus* (Gagkaeva et al. 2020). Also, in the Central and Northwestern European regions of Russia, *M. nivale* is predominant and accounts for 64.7% of the analyzed strains from this territory. In 2020, *M. seminicola* M. Hern.—Restr., Seifert, Clear & B. Dorn was first discovered in Russia (Gagkaeva et al. 2020). *M. seminicola* causes symptomless endophytic infections in cereals, and, from an ecological perspective, additional information about the distribution of this species would be essential. In the southern European region of Russia that is rarely affected by snow mold disease (Ivaschenko et al. 1997), *M. majus* and *M. nivale* were equally represented in cereals. On the contrary, in the central part of Russia (Tatarstan Republic) with sufficient snow cover each year, a regular annual occurrence of snow mold in winter rye was revealed for the period 2001–2017 (Ponomareva et al. 2019). The weighted average prevalence of snow mold in this period was 19.2%, which is near the value that is accepted for a disease being an epiphytotic (20%). In the Kirov region, *M. nivale* annual disease spread in winter ranged from 29 to 100% of the total growing area with a total loss of the cereal crops in nine out of 20 years (1999–2018, Sheshegova 2015; Utkina et al. 2019).

In the 1970s, snow mold was thought to be a disease that affected cereals only in those regions where plants were affected by prolonged (> 100 days) snow cover. For example, the northern areas of winter rye cultivation in Russia are characterized by long periods of humidity in autumn and early spring, higher snow cover, and frequent changes of snowy and thawing periods in winter, which induce fungal development (Glinushkin et al. 2018). However, today *Microdochium* species are seen as facultative snow mold pathogens that can also develop during the whole growing season of host plants (Matsumoto and Hsiang 2016) and produce symptoms of seedling blight, foot rot, leaf blight, and head blight.

Snow mold resulting from *M. nivale* infection is one of the most serious diseases of winter cereals (Tronsmo et al. 2001; Ergon et al. 2003; Prończuk et al. 2003; Ren et al. 2015), forage and turf grasses (Årsvoll 1973; Grosch and Schumann 1993; Abdelhalim et al. 2016; Stricker et al. 2017) in temperate and cold climatic zones. Mycelium, conidia, and ascospores are the infectious causes of *M. nivale*. No efficient crop protection strategy exists against these pathogens. Fungicides used so far are not environmentally friendly and many of them were recently prohibited in Europe (Gołębiowska and Wędzony 2009; Nielsen et al. 2013; Dyda et al. 2019). Also, it is impossible to spray fungicides under snow cover. The only chemical control mechanism available is seed dressing with fungicides that protects the germinating seed from seed-borne, but often not soil-borne inoculum. Thus, substantial effort must be invested in resistance breeding. However, the resistance of the most important cereal cultivars to this pathogen is not satisfactory or, in many cases, not existing. Rye is the hardiest cereal (Schlegel 2013; Fowler et al. 2014); however, it is also grown in Finland and in the northern zones of Russia, where winter wheat does not survive. Thus, rye must endure very low temperatures in winter periods with a long and high snow cover and is, therefore, prone to snow mold infections.

In this review, we concentrate on snow mold induced by *Microdochium* species that are harmful for all winter cereals. The focus is on rye, however, when results are missing, we also refer to winter triticale and winter wheat. The other winter cereals oat and barley do not play a role in Russia. In Central Europe, where winter barley is extensively grown, the occurrence of damaging snow mold is rare.

## Taxonomy, host range, and economic importance

*Microdochium nivale*, a filamentous ascomycete that is haploid in its vegetative state, was firstly described by the Swedish mycologist E.M. Fries (1825) under the name *Lanosa nivalis* characterized by its ability to attack wheat and grass under snow cover (Noble and Montgomerie 1956). Due to its similarity with *Fusarium* species, this fungus was later named *Fusarium nivale* Ces. ex Berlese & Voglino (Wollenweber 1931; Gerlach and Nirenberg 1982) and was re-classified in 1980 as *Gerlachia nivalis* (Ces. ex. Sacc.) W. Gams & E. Müll. (Gams and Müller 1980). Shortly after, Samuels and Hallett (1983) assigned the species to the genus *Microdochium*.

The recognition of two *varietas*, primarily based on the difference in conidial dimensions and septation, was firstly proposed by Wollenweber (1931) as *F. nivale var. nivale* and *F. nivale var. majus*. A recent examination of the *elongation factor 1 alpha* (*EF-1α*) gene sequence led to the re-classification of the two *varietas* of *M. nivale* as separate species, *M. nivale* (Fries) Samuels & I.C. Hallett and *M. majus* (Wollenw.) Glynn & S.G. Edwards (Glynn et al. 2005; Watanabe et al. 2011; Hayashi et al. 2014). *M. nivale* and *M. majus* can be differentiated by morphological means (Gerlach and Nirenberg 1982; Lees et al. 1995; Hernández-Restrepo et al. 2016), quantitative real-time PCR (Nielsen et al. 2013; Sonia et al. 2018), molecular markers (Hayashi et al. 2014; Matušinsky et al. 2019), and gene-sequence analyses (Einax and Voigt 2003; Jewell and Hsiang 2013). Very recently, Abdelhalim et al. (2020) demonstrated that *M. nivale* isolates revealed genetic differences related to different host plants (grasses *vs*. cereals) and different geographic regions (Norway and UK *vs*. North America) based on gene sequencing. This supports the assumption that some host specialization exists among *M. nivale*.

The genetic differences between *M. nivale* and *M. majus* are poorly characterized since their genome sequences are absent in public databases. However, the genome structure (3589–3816 Mbp size, 7617–8080 of annotated genes) of these species was briefly described by Jewel (2013). The only *Microdochium* species for which the genome sequence is available is *M. bolleyi* (David et al. 2016).

Usually one of the *Microdochium* species is prevailing in a crop (Nicholson et al. 1996). *M. majus* has a higher prevalence than *M. nivale* in most years in all cereals except rye, in which *M. nivale* was found to be more common. This is substantiated by a study of Danish cereals where *M. majus* has been more common than *M. nivale* for the last 50 years in wheat, barley, and triticale (Nielsen et al. 2013). In a study from the UK, 64% of infected samples involved *M. majus* and 36% *M. nivale* in wheat (Doohan et al. 1998). Also, in spring cereals, both species were identified at significant amounts onto the seed and in seedlings (Nielsen et al. 2011; Jonavičienė et al. 2016). They reported an advantage in the fitness of *M. majus* over *M. nivale* in spring wheat, spring triticale and spring oats, whereas spring barley was more sensitive to *M. nivale*. In contrast, McNeil et al. (2012) found on the seed of spring oats almost exclusively *M. nivale* whereas in spring barley seed *M. majus* dominated. Real-time PCR confirmed the presence of both pathogens in the seedlings of all cereal species tested (McNeil et al. 2012). Simpson et al. (2000) reported that *M. majus* showed a selective advantage on winter wheat and winter oat seedlings and *M. nivale* showed a strong selective advantage on winter rye seedlings in a mixed inoculation trial. Cockerell et al. (2009) suggested that spring wheat and oats are at risk from high levels of *Microdochium* infection, and spring barley is also at risk but only at levels exceeding 30% seed infection. All studies agree that winter rye (*Secale cereale* L.) is mostly colonized by *M. nivale* (Płażek et al. 2011; Żur et al. 2011; Pociecha et al. 2013a).

Economically, snow mold is important in all northern countries. The presence of a prolonged snow cover and moderately warm temperatures from February to April contribute to the spread and development of the disease on a large territory of Russia, leading to significant yield losses in fields cultivated with susceptible varieties. For example, from 2017 to 2019 snow mold affected an area of 445,600 to 856,500 hectares in Russia (Review 2020). Snow mold has become an important disease of winter wheat in Latvia during the last several years (Bankina et al. 2012).

## Symptoms and pathogenicity

*Microdochium nivale* and *M. majus* invade plants in late autumn or during the early winter period at low (near freezing) temperatures under dark and humid conditions of a snow cover where they proliferate and spread in the host tissues causing pink snow mold. *M. nivale* infects different parts and tissues of the plant both above and under the soil surface, starting from the endoderm and cortex of the root crown and then spreading to the epidermis of the leaves and leaf sheaths (Pociecha et al. 2013b). During infection, infectious hyphae penetrated directly into the cell wall of the epidermis with the help of a penetrating pin and then quickly spread in the host tissues both intercellularly and intracellularly (Kang et al. 2004). In this study, no entry of the host tissues via stomata was observed. Dubas et al. (2011) determined that the infection in triticale was by hyphal growth starting at soil level, which then passed into the leaves of the plant, but this penetration occurred only through the stomata and was followed by the formation of haustoria, resulting in intracellular growth. Żur et al. (2011), observing an infectious process of *M. nivale* on rye, also found that numerous hyphae penetrated the leaves through stomata within days following inoculation. Mycelium grew out of the soil and gradually penetrated the cells of the cortex and vascular tissues and then spread out. As such, both studies agreed that *M. nivale* infection began at ground level and progressed vertically up the plants before entering the leaves via stomata.

**Fig. 1:**
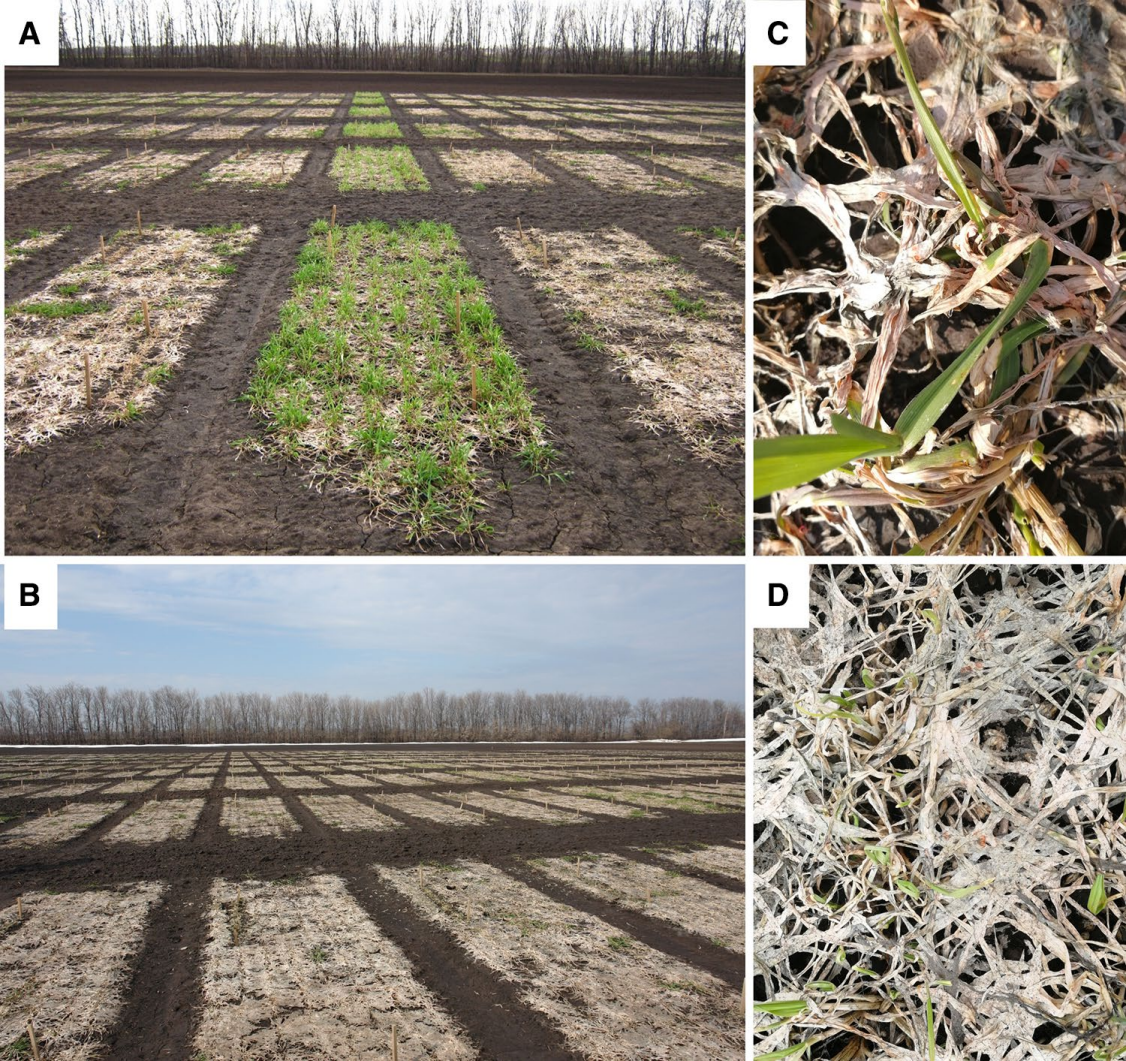
Natural snow mold infection of winter rye in Tatarstan/Russia. **a** Yield plots of winter rye with a snow mold-resistant entry in the middle (April 2018); **b** the susceptible winter rye varieties in conditions of severe snow mold infection (May 2019); **c**, **d** Details of snow mold-infected plots with some surviving plants (May 2019)

Microclimatic conditions, especially the temperature and humidity of the environment, strongly influence the infection process (Smith 1981; Hömmö 1994; Sugiyama and Shimazaki 2007). Immediately after snow melt, the fungi show a pale pinkish mycelium in patches (Fig. 1) and/or orange sporodochia. After drying, the dead leaves form a compressed paper-like layer. The main effects of snow mold attack are a reduction of seed germination by seed-borne inoculum (Hudec and Muchova 2010) and pre- and post-emergence death of seedlings leading to a significant reduction in grain yield (Humphreys et al. 1995). Since *M. nivale* can survive in the soil saprophytically for many years, inoculum will be available to attack crops despite a long absence of snow (Tronsmo 2013).

Both *Microdochium* species are not only causing pink snow mold symptoms under cool and humid conditions during spring and fall (Abdelhalim et al. 2020), but also seedling blight, foot rot (Clement and Parry 1998; Amein et al. 2007; Walker et al. 2009; Nielsen et al. 2011; Jørgensen et al. 2012), and head blight (Xu et al. 2007), the latter resembling the symptoms of *Fusarium* head blight (Gorkovenko et al. 2009; Gagkaeva et al. 2019). Late head blight infections also lead to seed-borne inoculum that could be a great problem in cereal stands for seed multiplication. In 2016, numerous reports on head blight and necrotic lesions on the flag leaves of winter wheat were observed in the Southern European region of Russia induced by both *Microdochium* species as shown by DNA analyses (Gagkaeva et al 2017). An extended period of wet and cool conditions might replace a long-lasting snow cover. Thus, *Microdochium* infections may become even more important with global warming, as they may prevail under conditions of large fluctuations in climatic variation and less seasonal predictability.

Miedaner et al. (1995) described the colonization of rye plants by *M. nivale* during the whole growth period by a species-specific enzyme-linked immunosorbent assay (ELISA). The highest fungal protein content was found in the plants directly after snow melt. During further rye growth, protein content decreased substantially, but showed a second peak between anthesis and full maturity. At milk ripening a foot rot rating of 3.3 on the 1–9 scale was recorded. This clearly demonstrates that *M. nivale* is capable of damage also at later growth stages. Interestingly, there was no correlation between young and adult plant stages for the resistances of 12 inbred lines illustrating that resistance to snow mold and *M. nivale* induced foot rot are attributed to different defense mechanisms. Similarly, Maurin et al. (1995) found a moderate, but non-significant correlation (*r* = 0.358) between foot rot and head blight resistance in winter wheat.

Both *M. nivale* and *M. majus* can reproduce asexually and sexually (Litschko and Burpee 1987; Lees et al. 1995; Parry et al. 1995), and the teleomorph belongs to *Monographella*. In a study of *M. nivale* isolated from grasses and cereals in Canada, most strains did not produce perithecia in vitro (Smith 1983). Some cereal strains formed these structures more frequently than others, but most of the perithecia did not mature. Mating type genes and reproductive strategy in *M. nivale* fungi are not yet fully understood. Fertility can be disturbed in many ways not only by genetic factors but also by environmental factors such as temperature, light, humidity, substrate, and physical factors that can affect different stages of development or the function of perithecia (Matsumoto and Hoshino 2008).

It is extremely important to note that *Microdochium* species do not form secondary metabolites toxic to mammals and humans (Edwards 2004; Glynn et al. 2005; Kabak et al. 2006; Xu et al. 2007) and accompany as endophytes plants throughout their life cycle.

## Measures for controlling snow mold

For the control of snow mold, agronomic measures, fungicides, and biological control have been examined. Also, sowing time, fertilizer application, and residue management can be used to manage snow mold. It is important that the plants are not developed too far when snow arrives, because dense plant stands can be faster colonized by the fungi.

Crop rotation is another important measure for control. Inclusion of spring annual or non-host crops in rotations leads to a rapid decrease in snow mold disease potential, probably due to reduced inoculum density in soil and changes in soil microbe populations (Murray et al. 1999; Gossen et al. 2001). In addition, after crop rotations with legumes such as alfalfa, sweet clover, or pea, less snow mold occurs on wheat, but with each subsequent sowing of winter wheat its severity increased (McKey and Reader 1953).

Concerning fertilizer, Dempsey et al. (2014) demonstrated phosphate (PO_3_^3−^)-mediated inhibition of *M. nivale* and *M. majus*. In vitro experiments have shown a direct inhibitory effect on the mycelial growth of both species. Inhibition of mycelial growth *in planta* would allow increased time for initiation of host defenses, and the combination of direct and indirect effects is likely to contribute to the reduced susceptibility of some genotypes. However, the application of PO_3_^3−^-containing fertilizers in the field has to be examined in greater detail.

Autumn-sown cereals are generally seed-dressed with fungicides in Northern Europe, thus reducing the risk of seedborne *M. nivale*. The attack of soilborne inoculum is reduced only for the first days of germination, but not in later stages of seedling development. The challenge of fungicide application is related to the fact that the activity of the pathogens occurs in the period when direct treatment of plants is difficult or impossible. Since the pathogen can reduce germination and also attack at later stages of plant development, a greater risk of plant losses by seed-borne inoculum occurs in organic farming (Johansson et al. 2003).

Numerous chemical protective agents used to combat both pathogens are available varying in their uptake and biochemical mode of action. The activity of the pathogen is inhibited by intervention in fungal mitosis, reduction of respiratory enzyme activity, inhibition of sterol, DNA and RNA synthesis, restriction of amino acid uptake, or inhibition of ATP production (Smiley et al. 1992; Yang et al. 2011).

Fludioxonil is highly effective for controlling *M. nivale* and *M. majus* by seed treatment (Glynn et al. 2008; Walker et al. 2009). However, resistances to strobilurines have already been reported in the same studies. Higher contents of *M. majus* and *M. nivale* DNA were identified in seedlings emerged from untreated seeds compared with the seedlings emerged from fludioxonil-treated seeds (Jonavičienė et al. 2016). Reports on the effect of tebuconazole were inconsistent (Simpson et al. 2001; Walker et al. 2009; Jonavičienė et al. 2016).

Much research has been devoted to the development of alternative, more environmentally friendly methods, such as the use of natural biocontrol agents (Compant et al. 2005; Haas and Défago 2005; Palazzini et al. 2013). Among them, the usage of *Pseudomonas brassicacearum* MA250 (Levenfors et al. 2008; Andersson et al. 2012), *Acremonium boreale* (Smith et al. 1989), and two strains of *Bacillus sp.,* YKT 2–17 and MGD-11 (Hoshino et al. 2004), were successful. Alternatively, the use of the nematofauna (mycohelminths *Aphelenchoides saprophillus* Franklin*, Paraphelenchus tritici* Baranovskaya*, Aphelenchus avenae* Bastian) for decreasing the damage on winter wheat infected with pink snow mold was analyzed (Tkachenko et al. 2015). Applied in autumn, a water suspension based on *A. saprophillus* on winter wheat in a dose of 160,000 individuals m^−2^ reduced disease incidence in spring threefold, thus increasing grain yield by 14.4%.

In conclusion, the best measures to limit snow mold damage in winter cereals are to regulate the nutrition of the plants increasing their winter hardiness, crop rotation, chemical seed treatment, and the introduction of resistant varieties (Murray et al. 1999).

## Physiological factors affecting infection and symptom expression

In general, a stable snow cover on unfrozen soil provides insulation, darkness, and humidity, creating favorable conditions for the snow molds (Bruehl and Cunfer 1971). These conditions reduce competition with other soil-borne pathogens that require higher temperatures (Hsiang et al. 1999; Chang et al. 2006) and limit the photosynthetic activity of the plant, thus weakening its protective responses (Murray et al. 1999; Matsumoto 2009). Reduced photosynthetic activity also limits the resources available for the pathogens resulting in increased interspecific competition (McBeath 2002). Although the inoculum density of pathogens is high in many growing regions, the environmental conditions required for disease development are much less predictable and consistent. Many other factors contribute to winterkill of cereal stands, including freezing conditions without snow.

The specific environmental conditions that contribute to the disease make it extremely difficult to reproduce experiments for evaluating resistance to snow mold (Kruse et al. 2019). Most of the studies on snow mold resistance used cold acclimation as a pre-treatment. The maximal resistance was detected exclusively in cold-hardened plants (Miedaner et al. 1993; Hömmö 1996; Laroche et al. 1997), but genotypes differ in their ability to obtain cold-induced resistance (Tronsmo, 1994; Pulli et al. 1996; Gołębiowska and Wędzony 2009; Kondo et al. 2012). It was shown that cold acclimation is necessary for the initiation of the defense system against snow mold infection (Nakajima and Abe 1996; Ergon et al. 1998; Tronsmo et al. 2001; Gołębiowska and Wędzony 2009; Płażek et al. 2011). According to Gaudet and Kozub (1991) and Gaudet (1994), there is no direct correlation between snow mold resistance and frost tolerance, but cultivars with high frost tolerance often show higher persistence in response to snow mold. On the other hand, young plants already killed by frost are, of course, not able to show any snow mold resistance. Thus, no strong correlation was found between snow mold resistance and frost tolerance in winter wheat (Bruehl 1982; Gaudet and Kozub 1991) and perennial ryegrass (Hofgaard et al. 2003). Recovery after pathogen attack depends on the survival of crowns, which are the reservoir of soluble carbohydrates used for regrowth after stress cessation.

In winter cereals, snow mold severity interacts with winter hardiness or frost tolerance in a complex way. Several physiological traits are suspected or known to influence snow mold resistance (SMR) in winter cereals including plant size (Bruehl and Cunfer 1971), carbohydrate accumulation and metabolism (Kiyomoto 1987; Mohammad et al. 1997; Kawakami and Yoshida 2012; Yoshida and Kawakami 2013; Meguro-Maoka and Yoshida 2016), and the regulation of genes for defense-related proteins, transcription factors, and kinases during the cold-hardening process (Gaudet et al. 2011; Gaudet and Laroche 2013). However, the relationship between frost tolerance and snow mold resistance is not well understood. Evidence suggests that they are not controlled by the same genes for winter cereals, although some common physiological processes might be involved. When plants are exposed to one stress factor, this can change their reaction to another stress factor, either biotic or abiotic. For example, freezing stress generally increases the susceptibility of plants to fungal attack and, on the contrary, infection of the plant by pathogens during winter reduce their resistance to frost.

Besides these effects, temperature also plays a crucial role for the fungi. *Microdochium* species are usually cited as a common cause of cereal diseases in cooler regions (Daamen et al. 1991; Nakajima and Abe 1994; Tronsmo et al. 2001; Bertrand et al. 2009). This could be attributed to the dramatic inhibition of their in vitro growth at temperatures > 25 °C. This was confirmed from other studies in European countries that showed that *M. nivale* isolates grew optimally at 20–25 °C (Brennan et al. 2003; Doohan et al. 2003) or even < 20 °C (Hudec and Roháčik 2003). In synthetic media, the fungi can grow at temperatures as low as − 6 °C and in the field at temperatures below 0 °C (Hömmö 1994; Ergon et al. 2003).

## Candidate genes and genomics of host resistance

Snow mold resistance (SMR) is a complex quantitative trait manifesting as a continuous distribution of disease response that is highly influenced by environmental conditions (Kruse et al. 2017). Further, like other complex quantitative traits, SMR is conferred by numerous quantitative trait loci (QTL) and influenced by genotype × environment interactions since the environment affects not only host resistance but also the composition of the pathogenic complex that causes the disease.

A potential role in defensive response to *M. nivale* infection was proposed for catalase, peroxidase, and thiol-specific antioxidant proteins (Gołebiowska-Pikania and Golemiec 2015) in winter triticale. Recently, Gołebiowska et al. (2019) concluded that genotype and cold treatment significantly affected the amount of small proteins in winter triticale leaves. The pattern of these proteins could be correlated with the level of the resistance to pink snow mold infection. Four cold-accumulated proteins: ADP-binding 38 kDa resistant protein, β-ATP synthase subunit, as well as 25 kDa and 49 kDa thioredoxin peroxidases, can play a role in preparing the plant for the attack of a fungal pathogen in a resistant winter triticale, maintaining the redox balance and energy pathway in the seedling leaves. The research carried out by the authors can point to molecules for further analysis of the *M. nivale* pathogenesis process in winter cereals.

Nevertheless, these proteins are only a part of a complex mechanism that is still not fully understood yet. Kuwabara and Imai (2009) proposed a model of a signalizing network for disease resistance acquired through cold acclimation that includes three pathways: (1) defense pathways mediated by salicylic or jasmonic acids, (2) cold acclimation by cold-binding transcription factors, and (3) novel, still unknown pathways. Moreover, disease resistance is also related to physiological adaptation that leads to the accumulation of storage carbohydrates during cold acclimation (Gaudet et al. 1999). According to the findings of Płażek et al. (2011), a link between the resistance to *M. nivale* and a higher content of total carbohydrate concentrations in inbred lines of winter rye exists. Żur et al. (2013) demonstrated that chitinases are actively involved in the response of triticale plants to *M. nivale* infection. The activity of both studied glucanhydrolases is suppressed during the cold, possibly as a consequence of an altered metabolism. Thus, the total activity of chitinases in plants might be indicative for the plant’s resistance/susceptibility against a fungal pathogen.

Szechyńska-Hebda et al. (2016) have shown that the cellulose in the cell walls of resistant triticale varieties after cold treatment has a more compact and integrated structure with thicker and longer fibers than that of the respective susceptible variety. This structure of cellulose limits water sorption, promotes stronger binding of crystallization water with macromolecules, prevents depolymerization during decomposition, and ultimately leads to the higher thermal stability of the cell wall. Besides these detailed studies, the mechanisms of resistance to pink snow mold during winter are far from being understood.

As a tool for further dissecting SMR, genomic data could be extremely helpful. In the first study on QTL mapping in winter triticale, QTL associated with resistance components to *M. nivale* was detected on chromosomes 1B, 2A, 3A, 3B, 5A, 5B, 6A, 6B, and 7B in climate-chamber tests (Szechyńska‐Hebda et al. 2013). Surprisingly, no rye chromosome carrying SMR loci was found in this study. However, this might be explained by the source of the rye genome used for the primary triticale crossing.

Also, in winter wheat, there have only been limited reports on QTLs for SMR. Kruse et al. (2017) detected two QTLs. One of them was associated with both freezing tolerance and SMR on chromosome 5A. This QTL was closely linked with the *Fr-A2* (*Frost-resistance A2*) locus. The association of both traits may have resulted from pleiotropic effects or from the fact, that higher tolerance to low temperatures enabled the plants to better defend themselves against snow mold.

Lozada et al. (2019) reported the first major study on the genomics of SMR by using genome-wide association study (GWAS) and genomic selection (GS) approaches in winter wheat. This research identified genomic regions in diverse populations of US winter wheat lines from the Pacific Northwest region (PNW). A total of 14 linked snow mold resistance markers were identified on chromosome 5A. Some of these significant SNPs co-localized with the freezing tolerance *Fr-A2* locus and others coincided with the major vernalization gene *Vrn-A1*. Additional SNP markers on chromosomes other than 5A and 6B were identified that have not been reported elsewhere, and hence may represent novel QTLs affecting snow mold tolerance in PNW winter wheat. Nevertheless, detected regions on chromosomes 5A and 6B may be syntenic between wheat and triticale and could give a good start for investigations in other related cereals crops, e.g., rye.

Kovi et al. (2016) conducted a major study of whole transcriptome sequencing (RNA-seq) in order to understand the response of *Lolium perenne* to an early infection of *M. nivale*. Transcriptomic changes in perennial ryegrass during early infection were detected. Some of the differentially regulated genes were found to be disease-related proteins, calmodulin-binding proteins, lipid transfer proteins, and flavonoid biosynthesis. The list of suspected candidate defense associated genes and cell morphogenesis associated genes identified in this study may provide a good scientific basis for further research toward understanding the host–pathogen interactions and describing the molecular mechanisms of SMR.

For dissecting individual components of SMR and to accelerate breeding progress in winter rye, research on genomic tools should be prioritized. Generally, genomic resources in rye are limited as recently reviewed by Miedaner et al. (2019). Shortly, there is one inbred line (Lo7) where recently a whole-genome sequencing was finished and medium- to high-density SNP assays are available. Alternatively, DArTSeq can be commercially performed in the rye. This suffices for all marker-based methods; however, the availability of functional gene sequences is very limited. Genome-wide association studies (GWAS) would allow to screen large numbers of genotypes. For selected, highly effective resistance sources, also a bi- or multi-parental QTL mapping could be taken into account, because this allows the localization of the respective genomic regions with higher precision. These experimental approaches could be combined for gaining a broad training population for genomic selection (GS) procedures. Due to the complex host–pathogen interaction and the quantitative nature of SMR, this technique could play a key role in improving the resistance in winter cereals as shown by many examples of other diseases caused by hemi-biotrophic fungi.

Combining GS with phenotypic selection will increase the genetic gain by selecting lines with improved tolerance to winter freezing and SMR based on genomic-estimated breeding values. In addition, progress could also be achieved by analyzing the transcriptome profile of susceptible vs. resistant winter rye inbred lines during different stages of host colonization by *Microdochium*. This tool will serve to characterize early signaling events during the host-snow mold interaction and could result in a list of candidate genes crucial for resistance reaction. By the combination of genomic and transcriptomic data, candidate genes for SMR could be verified more effectively in order to overcome the inherent weaknesses of each individual method. In rye, however, no such studies were conducted till now.

## Testing and breeding for snow mold resistance

In cereals, a quantitative variation exists among cultivars for SMR with relatively high heritability that is conditioned by the additive effects of many loci (Iriki and Kuwabara 1993). Breeding for SMR faces several challenges. In the field, the length of snow cover is not predictable, other snow mold fungi might interact even when *M. nivale* or *M. majus* are inoculated, and in each year the overwintering conditions greatly vary. Moreover, injuries due to snow mold can hardly be differed from other causes of winter damage, e.g., ice encasement, carbohydrate depletion, low photosynthetic efficiency (Ergon et al. 1998). Therefore, to identify SMR techniques are needed for use under controlled conditions mimicking the snow cover with high precision (Gaudet and Bhalla 1988). Additionally, *M. nivale* isolates may lose their pathogenicity rather soon when cultivated on laboratory media (Johansson et al. 2003).

**Fig. 2:**
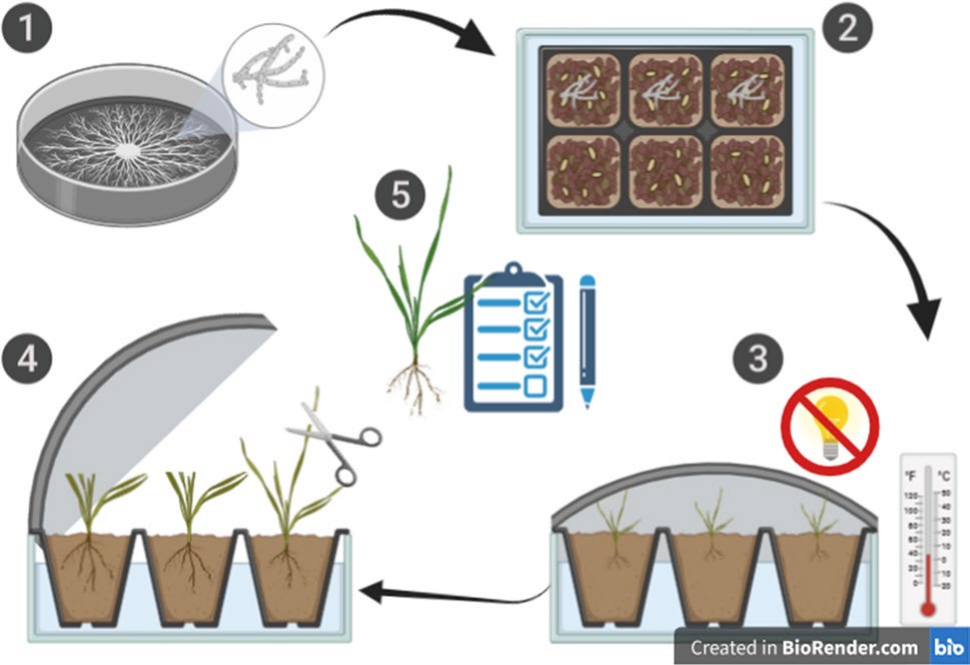
Detailed scheme for testing SMR in climate chambers: (1) Mycelium was grown on Potato Dextrose Agar (PDA) for 1–2 weeks at 19 °C in darkness. (2) Mycelium was transferred to a sterile mixture of soil, peat, and sand (3:1:1 by volume), containing 5% w/w of ground wheat grain, and cultured for 14 days; inoculum was gently mixed and spread onto the soil of each plant. (3) Imitation of the conditions occurring under the snow cover by covering the plants with moist (watering) cellulose tissue paper (blotting paper) and a black plastic bag, placed in a cold chamber at 0–2 °C in darkness for 5–8 weeks. (4) The plants were cut at 5 cm above the soil surface and allowed to regrow for 3 days at 12 °C in 100 μmol m^−2^ s^−1^ PPFD light and next for 7 days at 18 °C in 250 μmol m^−2^ s^−1^ PPFD light. (5) Estimation of Average Regrowth Index (ARI)

Under controlled conditions of climate chambers, in which plants are exposed to low temperatures for hardening, inoculation, and incubation, tests could be conducted throughout the year (Sunderman 1964). The degree of the disease can be controlled by varying the duration of incubation at low temperatures. The temperature should be adjusted to the cereal species. Rye, for example, needs lower temperatures (− 4 °C) to stop vegetative growth than wheat (Schlegel 2013). The optimal conditions for the development of snow mold on winter wheat in a controlled environment have been identified by Gaudet and Kozub (1991) and are presented in Fig. 2.

Preparation of *M. nivale* inoculum for testing winter cereals was described by Prończuk and Prończuk (1987). A pre-hardening of test plants is highly important for the outcome of the test (Miedaner et al. 1993). Plant regrowth was evaluated using the Average Regrowth Index (ARI), i.e., a visual rating scale from 0 to 5, where ‘0’ denotes a plant without any visible symptoms of infection and ‘5’ a completely dead plant with no signs of leaf elongation.

According to Hömmö (1994), the results obtained in the field were closely correlated with those obtained in the climate chamber. Jamalainen (1974) reported that some results in the snow mold chambers are in conflict with those in the fields. Accordingly, Miedaner et al. (1993) found significant, but moderate correlations (*r* = 0.51–0.57, *P* < 0.01) between growth-chamber results and a 2-year field test across 21 rye inbred lines. Because of its utmost importance for breeding, this correlation should be investigated with larger populations.

The breeding methods currently deployed for the identification of SMR in winter cereals in Russia are based on phenotypic screening in the field especially since natural conditions are predisposed to evaluation. For breeding programs in a number of other European countries, evaluation in climate chambers are used. However, a combination with field trials should be most advantageous.

Identifying resistance sources and visual selection for SMR has been proved difficult, because of the close interaction of the overwintering ability of the host and quantitative host resistances (Murray et al. 1999). To select for entries with high winter hardiness, SMR, and additionally favorable agronomic traits, especially grain yield, different progeny tests are necessary: (1) Freezing tests at − 10 °C to − 23 °C depending on the cereal species to select for tolerance to sub-zero temperatures without snow cover, (2) climate-chamber tests for SMR at 0 °C to + 2 °C with simulation of a snow cover, and (3) field tests at several locations with/without regular snow mold incidence. Multi-environment tests are absolutely necessary, because the winter conditions, esp., length of snow cover and freezing temperatures, cannot be predicted and even the more resistant progenies will suffer when a sufficiently strong attack of snow mold occurs (Gaudet 1994; Nissinen 1996; Matsumoto and Hoshino 2013).

For assessing SMR in practical breeding, several indicators that characterize the degree of resistance have been successfully applied. Firstly, breeders use the estimation of regrowth, after artificial or natural infection. Average Regrowth Index (ARI) was calculated according to the formula: [(*n* × 0) + (*n* × 1) +⋯+ (*n* × 5)] × *N* − 1, where: *n* = number of plants corresponding to each disease rank (0–5), and *N* = total number of observations. The lower the ARI value, the higher the resistance of the plants to the pathogen. Secondly, tillering is considered as an important resistance trait. The number of infected tillers after snow melt compared to the number before overwintering is a successful indicator of the selection of resistant lines. Finally, an extremely important trait is the yield of an entry after *M. nivale* attack, which ultimately shows the genetic ability to withstand an attack by *M. nivale* infection, especially in years with a strong fungal development. Besides these direct traits for assessing SMR, an important secondary trait is the time till maturity, because a high infection rate slows down further cereal growth. Susceptible varieties may come 1–2 weeks later to maturity than resistant varieties.

In breeding programs aimed at increasing the level of SMR, the availability of effective resistance sources is crucial. An early study of SMR in the US Pacific Northwest showed that out of 12,000 wheat samples collected from around the world, ten quantitatively resistant samples were the only lines statistically identified as resistant (Bruehl 1982). This screening resulted in the development of the Sprague variety with a useful level of quantitative resistance to pink snow mold. SMR among winter wheat cultivars from other countries also has been reported (Amano and Osanai 1983; Gaudet and Kozub 1991; Brennan et al. 2005; Serenius et al. 2005).

In rye, also various sources of resistance were identified by different authors (Table 1). Kobylyansky and Solodukhina (2012) found sources of SMR among wild species and varieties of cultivated rye growing in the mountainous regions of the Caucasus and the Alps on the border of permanent agriculture following Vavilov’s (1964) suggestion to search for resistance sources in the regions of the coevolution between host and pathogen. Alpine zones are characterized by the presence of a continuous snow cover over an extended period of time. Several authors showed that the number of sources with a durable resistance is very limited, especially in winter triticale. The work on the identification of new resistant varieties, which have not been previously used in breeding, was practically not carried out in recent years. The greatest number of winter rye varieties and lines resistant to snow mold have been found in the Federal Research Center N.I. Vavilov All-Russian Institute of Plant Genetic Resources (St. Petersburg) due to the engagement of the curators (see Table 1). It should be noted that there are no varieties that are not attacked by snow mold, and there is no systematic information on the inheritance of SMR resistance in the different sources. Therefore, the use of genetically diverse sources of resistance is indispensable for durable SMR in winter cereals. Since additive inheritance of resistance to *M. nivale* prevails, increasing SMR by recurrent selection should be successful.

**Table 1:** Summary data of resistance sources for *Microdochium nivale* in winter cereals

Cereal	Germplasm	Reference
Winter wheat	Eltan (PI536994), Bruehl, Masami, Stephens, Xerpha, Madsen, P.I.173438, P.I.172582, C.I.14106 (USA) Cubus (Germany), Folke, Sleipner, Renan, Arminda, Munstertaler, Saint-Johann (Sweden), Linna, Aura, Vakka (Finland) Imeni Rappoporta, Mironovskaya 808, Zarya, Moskovskaya 39 (Russia)	Kleijer (1988), Peterson et al. (1991), Hömmö and Pulli (1993), Kuwabara et al. (1996), Jones et al. (2001, 2010), Tkachenko et al. (2015)
Winter rye	***Varieties***—Tatarskaya 1, Estafeta Tatarstana, Zilant, Lisitsyna, Kaluga 45, Kamalinskaya 13, Taezhnaya, Kirovskaya 89, Vyatka 2, Dymka, Flora, Grafinya, Rosinka, Ilim, Falenskaya 4, Purga, Chulpan, Chulpan 3, Ilmen, Iset, Super Baby 2, Volna, Korotkostebelnaya 6, Kharkovskaya 88, Bezenchukskaya 88, Volkhova, ***S. cereale ssp. tetraploidum*** -Shatilovskaya tetra, Populyatsiya I-82 tetra, Sibirskaya krupnozernaya ***Accessions from VIR World Collection*** (N.I. Vavilov All-Russian Institute of Plant Industry, St. Petersburg): LAD-287, 1313, 11,349, 11,350, 11,357, 11,358 (Poland), Epos, Rerus (Germany), 11,330–11,334 (Sweden), Lassaer (Austria) Kevsole, Kefermarkter, Edelhofer, Toivo, 11,363, 10,953 (Finland), Münstertaler, Haunsberg, Niederndorferberg (Switzerland) Feniks (Belgium), 11,385 (Yugoslavia), 11,150, k-11389 (Portugal), 11,306 (Argentina), 11,179, k-11180 (USA), 11,388 (Tajikistan), 11,398 (Georgia), 11,131 (Azerbaijan), Belta tetra (Belarus), Beve, Borotba (Ukraine) 11,340 (France)	Tkachenko et al. (2015), Utkina et al. (2017), Ponomareva et al. (2019)
Winter triticale	Hewo, Modus (Poland) Sv 856,003 (Finland)	Gołębiowska and Wędzony (2009), Hömmö and Pulli (1993)

## Conclusions and further research needs

Snow mold is one of the most difficult to manage plant diseases due to several reasons. First, despite its big economic importance, snow mold is rather poorly investigated, because it was thought to be important only for those regions where snow cover is maintained for a long period. However, during the past decades *Microdochium* species appeared to parasitize not only during early spring under snow cover, but also during the whole lifetime of the plant resulting in foot rot, leaf blight, and head blight. These non-snow mold associated diseases of *Microdochium* spp. must be researched more intensively, because they occur also in regions without harsh winter. Also, the correlation of resistance among the different diseases must be analyzed. In *Fusarium* diseases, we had to learn that each plant growth stage resulted in a different resistance ranking of the genotypes. In addition, information on the correlation between resistance to *Microdochium* spp. and to other species of the snow mold complex is missing.

Second, although some varieties with quantitative SMR exist, most of the germplasm is highly susceptible to this disease, and only a small number of resistance sources is known in any winter cereal. Moreover, multiple testing environments are required for breeding since SMR is a complex quantitative trait that is highly dependent on environmental factors.

Third, given that the parasitic cycle of snow mold causing fungi proceeds mostly under the snow cover, spraying of fungicides is complicated or even impossible and, thus, the soil-borne inoculum cannot be controlled.

Fourth, snow mold-causing fungi, including *Microdochium nivale* and *M. majus*, are poorly characterized by molecular means. Knowledge on virulence factors as well as full genome sequences is missing, and even the molecular features of host infection are totally unknown. Finally, no robust and reliable experimental models for studying the mechanisms of host interactions with snow mold-causing pathogens are worked out. Phenotypic standardization is necessary because the disease manifestation after artificial inoculation of plants in the field or in vitro is highly affected by several environmental factors controlling both pathogen development and host resistance. For example, the exact growth stage and the nutritional status of the plants under snow mold attack are as important as the length of snow cover or the duration and peak temperatures of the freezing period without snow.

In practical breeding programs, the selection of SMR requires parallel testing for increased freezing tolerance and for artificial infection of *M. nivale* in separate climate chambers. At the same time, elite lines need to be evaluated in field trials in regions with a reliable snow cover. While selecting resistant genotypes, both a fast regrowth and a high grain yield after infection should be evaluated.

In future, reliable experimental models should be worked out for studying the molecular basis of pathogen virulence and plant resistance. In addition, priming of physiological resistance (e.g., conferred by cold acclimation or other treatments) should be preciously exploited as a way to control snow mold. The use of natural biocontrol agents might be promising since snow mold-causing fungi seem to be less competitive compared to pathogens thriving during the growth period, and so, the search and application of psychrophilic bacterial and fungal strains or nematode biotypes which protect plants could reduce the risk of crop loss.

Breeding progress can be accelerated by identifying new and effective resistance sources, by researching the genetic factors controlling host resistance, and by improving and standardizing screening methods. Due to the complexity of the resistance mechanisms, a poorly understood genetic background, and strong interaction with winter weather conditions, it is difficult to increase the resistance of winter rye only via conventional breeding methods. In future, modern genomics-based techniques should be applied to large plant populations with accessions of different origin to establish QTLs responsible for both SMR and freezing tolerance. On this basis, genomic selection procedures could be established, e.g., by estimating genomic breeding values for SMR with using important QTLs for freezing tolerance as fixed effects (Herter et al. 2019). Winter rye could play a key role in this research caused by its high genetic diversity as cross-pollinated species, the high freezing tolerance, and the availability of new, marker-based resources (Miedaner and Wilde 2019). In future, SMR will be an important trait for successfully growing winter cereals in areas of higher latitude, like in Finland, Russia, and Canada, in a more sustainable way.
